# The synergism of spatial metabolomics and morphometry improves machine learning‐based renal tumour subtype classification

**DOI:** 10.1002/ctm2.666

**Published:** 2022-02-20

**Authors:** Verena M. Prade, Na Sun, Jian Shen, Annette Feuchtinger, Thomas Kunzke, Achim Buck, Peter Schraml, Holger Moch, Kristina Schwamborn, Michael Autenrieth, Jürgen E. Gschwend, Franziska Erlmeier, Arndt Hartmann, Axel Walch

**Affiliations:** ^1^ Research Unit Analytical Pathology Helmholtz Zentrum München – German Research Center for Environmental Health Neuherberg Germany; ^2^ Institute of Pathology and Molecular Pathology University Hospital Zurich Zurich Switzerland; ^3^ Institute of Pathology Technical University Munich Munich Germany; ^4^ Department of Urology Technical University Munich Munich Germany; ^5^ Institute of Pathology, University Hospital Erlangen Friedrich‐Alexander‐University Erlangen‐Nürnberg Erlangen Germany; ^6^ Comprehensive Cancer Center Erlangen‐EMN (CCC ER‐EMN) Erlangen Germany

**Keywords:** machine learning, mass spectrometry imaging, metabolomics, morphometry, renal cell carcinoma, tumour of the kidney, tumour subtyping

Dear Editor,

Although mass spectrometry imaging generates both morphometric and metabolomics data, they have never been combined to improve machine learning‐based tumour typing. We demonstrate that the synergy of spatial metabolomics and morphometric data improves the classification of tumours of the kidney and thus has the potential to improve artificial intelligence (AI)‐based diagnostics.

Tumours of the kidney are a heterogeneous group of various types of cancer with characteristic histologic or genetic features that require tumour type‐specific therapies.^1^ Chromophobe renal cell carcinomas (chRCC) and renal oncocytomas – two tumour types that can sometimes be difficult to distinguish based on morphology alone – are associated with different prognosis, and the former has the potential to progress and metastasize.^2^, ^3^ Both immunoncological and targeted therapies are investigated; however, immunotyping and genotyping are laborious, fall short of standardization, and immunohistochemical markers have been shown to be unreliable.^4^, ^5^

We used matrix‐assisted laser desorption/ionization (MALDI) mass spectrometry imaging (MSI) because one of its greatest strengths is the ability to combine in situ mass spectrometric data with conventional histology or immunohistochemistry, making it a powerful and very useful tool for multiparametric high‐dimensional multi‐omics analyses.^6^, ^7^, ^8^, ^9^, ^10^ This potential has also been successfully applied for biomarker discovery and machine learning‐based renal tumour subtyping using unique molecular data.^11^, ^12^, ^13^, ^14^, ^15^

Our study was performed on a large patient cohort (*n* = 853, Table 1) and on clinically relevant FFPE tissue samples to distinguish clear cell renal cell carcinomas (ccRCC, *n* = 552), papillary renal cell carcinomas (pRCC, *n* = 122), chRCC (*n* = 108) and renal oncocytomas (RO, *n* = 71). For details about the MALDI imaging and the morphometrics analysis of H&E stained tissue sections, see the supporting information.

**TABLE 1:** Patient characteristics

**Patient characteristics**	*n* = 853
**Age range (median) (years)**	27–88 (65)
**Gender**	
Male	299 (40.4%)
Female	441 (59.6%)
**ISUP Grade**	
Grade 1	25 (4.1%)
Grade 2	241 (39.4%)
Grade 3	191 (31.3%)
Grade 4	154 (25.2%)
**Pathological stage**	
pT1	418 (55.8%)
pT2	90 (12.0%)
pT3	231 (30.9%)
pT4	10 (1.3%)
pN+	26 (15.0%)
pM+	4 (36.4%)
**Subtype**	
RO	71 (8.3%)
chRCC	108 (12.7%)
ccRCC	552 (64.7%)
pRCC	122 (14.3%)
Survival (dead/alive)	214 (31.5%)/465 (68.5%)
Overall survival median (months)	36

Abbreviations: ccRCC, clear cell renal cell carcinomas; chRCC, chromophobe renal cell carcinomas; pRCC, papillary renal cell carcinoma; RO, renal oncocytomas.

Morphometric features (*n* = 110) describing tissue or cell compartment colour, shape or size (Table S1) and untargeted metabolomic features (*n* = 2,111) were used for classifier training (Figure 1). Patients were randomly split into training (2/3) and independent validation set (1/3). The data were normalized for training and validation set, separately. Feature selection was done by calculating a Kruskal–Wallis test (*p* < 0.01) on the training sets. Details about the classifier training are in the supporting information.

**FIGURE 1:**
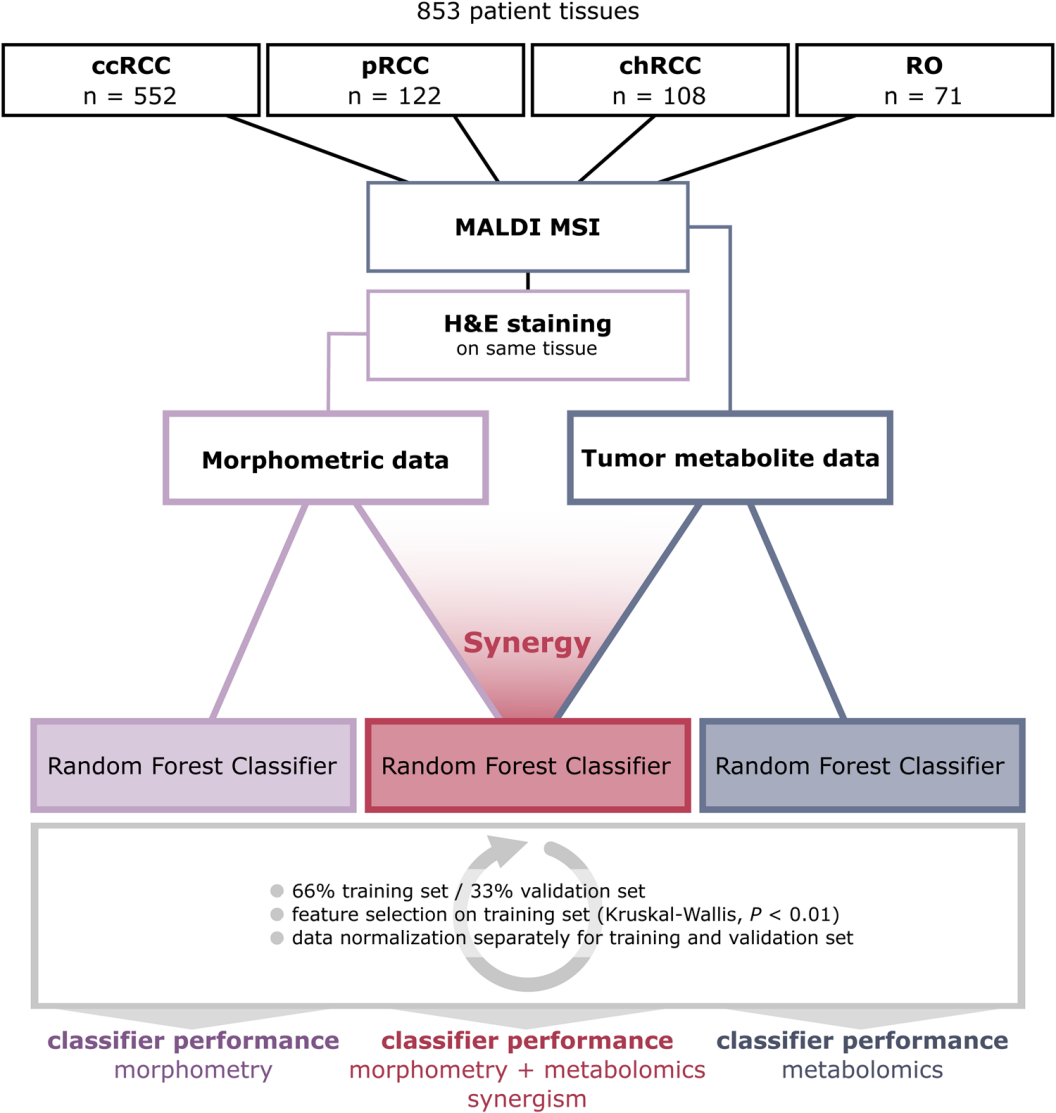
Workflow to analyze the synergistic effect of morphometric and molecular data on classifier performance. Eight hundred fifty‐three patient tissues were analyzed with MALDI mass spectrometry imaging (MSI), followed by hematoxylin & eosin staining and morphometric image analysis. Morphometric and molecular data of tumour regions were used both separately and in synergy to train three random forest classifiers. Patients were split into training and independent validation sets, followed by data normalization, feature selection and classifier training and validation

Using morphometric data alone, the classifier reached a mean accuracy of 77.81% (Figure 2A). It performed best for ccRCC (F1‐score: 86.22%) and worst for chRCC (F1‐score: 52.17%).

**FIGURE 2:**
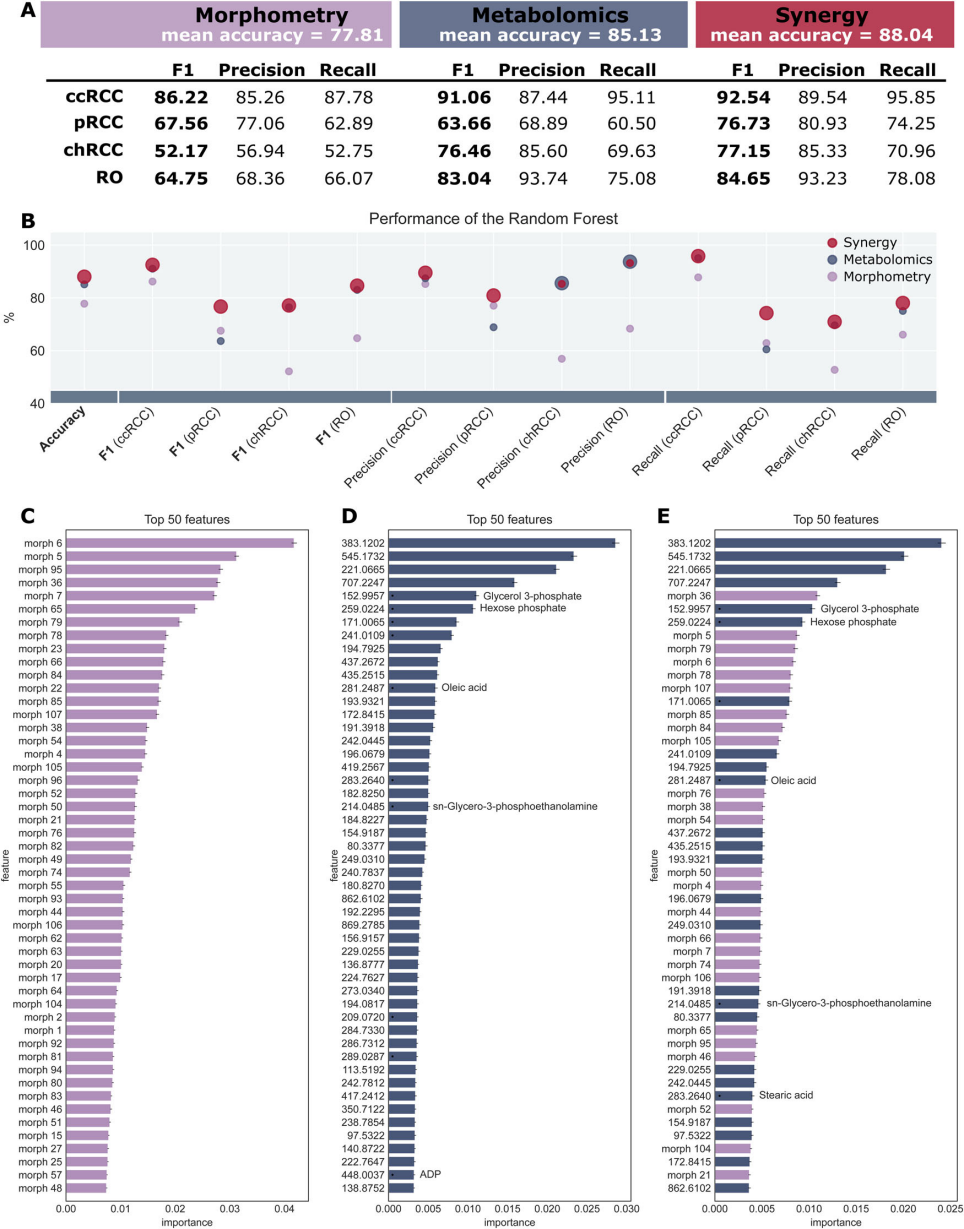
Synergistic effect of morphometric and molecular data on classifier performance on renal cell carcinoma subtypes. (A and B) Classifier performance of the random forests revealing a synergistic effect of morphological and molecular data. In (B), the best performing classifier is visualized with a larger marker. The synergistic effect is best seen for papillary renal cell carcinoma (pRCC), where the performance is improved by up to 10 per cent. (C‐E) Feature importance of the top 50 features for the random forest trained on morphometric data (C), metabolite data (D) and the synergy of both (E). In the latter, the top 50 comprise a mixture of both types of features

Trained on metabolomics data, the classifier reached a higher mean accuracy (85.13%) and performed even better on ccRCC (F1‐score: 91.06%), chRCC (F1‐score: 76.46%) and RO (F1‐score: 83.04%) (Figure 2A). Only on pRCC, this classifier did not perform as well as the classifier trained on morphometric data (F1‐score: 63.66%).

The third classifier was trained on the synergy of both data sets – morphometric data and molecular data – and outperformed the two previous classifiers for each tumour subtype (Figure 2A). It reached a mean accuracy of 88.04% and F1‐scores of 92.54% (ccRCC), 76.73% (pRCC), 77.15% (chRCC) and 84.65% (RO). When comparing each statistical measure, the synergistic classifier trained on both data sets almost consistently outperforms the other two (Figure 2B).

The synergy of morphometric and metabolite data not only improves general performance, but also seems to compensate for weaknesses of the two individual classifiers trained on either morphometric or metabolite data. For instance, the metabolomic classifier performed better compared to the morphometric classifier for ccRCC (F1‐scores: 91.06% vs. 86.22%), chRCC (F1‐scores: 76.46% vs. 52.17%) and RO (F1‐scores: 83.04% vs. 64.75%), while the morphometric classifier performed better for pRCC (F1‐scores: 63.66% vs. 67.56%). The classifier trained on the synergy of the two data sets outperforms either of the two (F1_ccRCC_: 92.54%, F1_pRCC_: 76.73%, F1_chRCC_: 77.15%, F1_RO_: 84.65%).

The Gini importance of each feature was calculated for the three classifiers, and the top 50 features were compared (Figure 2C‐E). The top 50 features from the classifier trained on both data sets represent an even mixture of morphometric and metabolomic features (Figure 2E). Example metabolite ion images of the top features in the classifier are illustrated in Figure S1. Interestingly, the four most important features are metabolites, and while half of the top features are morphometric, the importance of the best metabolite is twice as high as that of the top morphometric feature. This further illustrates the synergistic impact both data sets have on the classifier's performance.

Up to this point, the classifiers were trained to distinguish four different tumour subtypes, which is a much more difficult task than separating only two tumour subtypes. However, since ccRCC and pRCC are relatively easy to recognize by a pathologist using histology alone, we also tested our approach exclusively on the two remaining tumour subtypes – chRCC and RO (Figure 3A).

**FIGURE 3:**
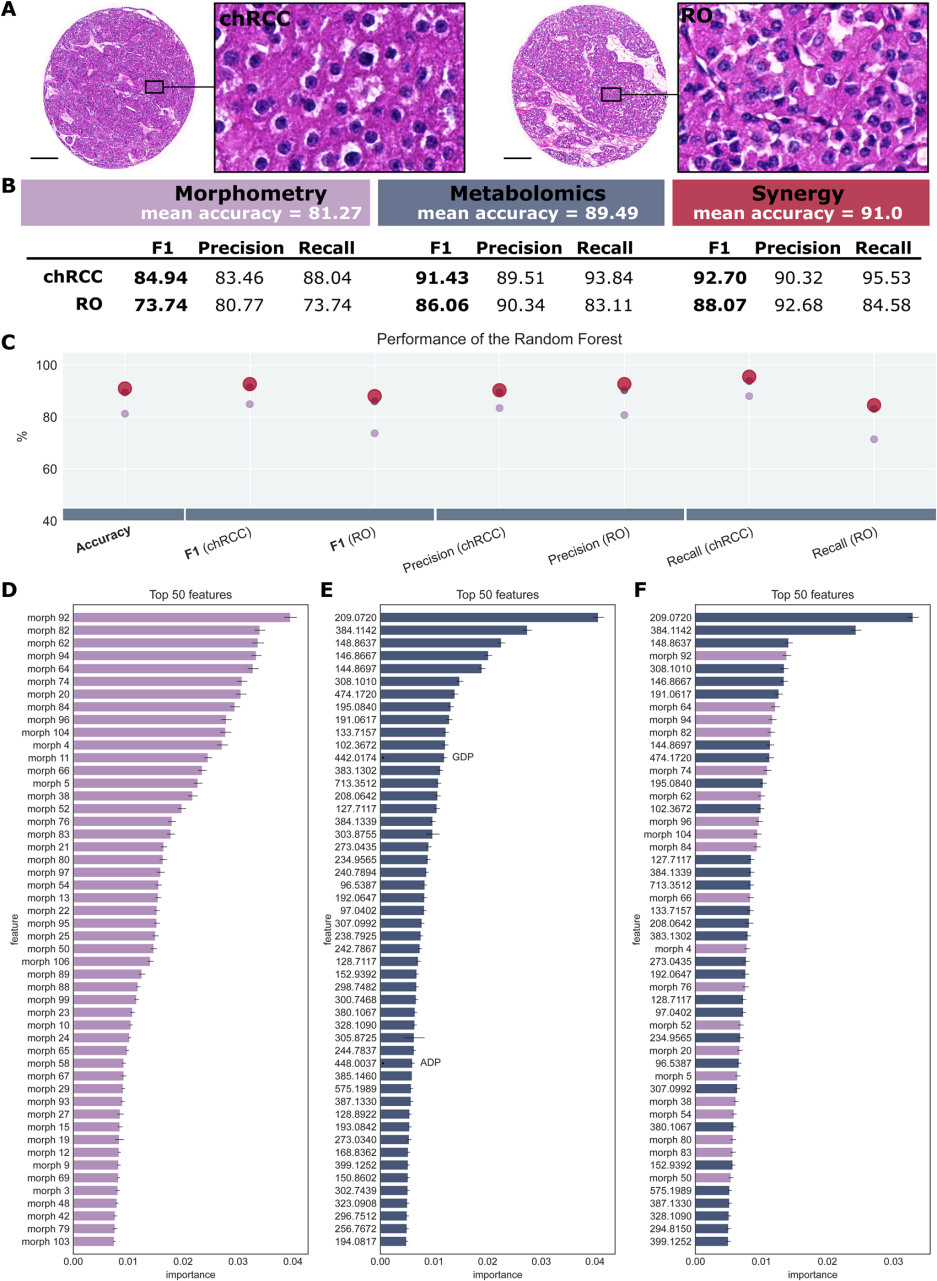
Synergistic effect of morphometric and molecular data on classifier performance of chromophobe renal cell carcinoma (ccRCC) and renal oncocytoma (RO). (A) Hematoxylin & eosin staining of chRCC and RO, illustrating their similar histology. (B and C) Classifier performance of the random forests revealing a synergistic effect of morphological and molecular data. In (C), the best performing classifier is visualized with a larger marker. (D‐F) Feature importance of the top 50 features for the random forest trained on morphometric data (D), metabolite data (E) and the synergy of both (F). In the latter, the top 50 comprise a mixture of both types of features. Scale bar: 200 μm

The same synergistic effect can be observed for the three classifiers (Figure 3B). On morphometric data, a mean accuracy of 81.27% is achieved, while on metabolomics data, the mean accuracy is higher with 89.49%. The synergy of the data further increases the accuracy to 91.0% with an F1‐score of 92.70% for chRCC and 88.07% for RO. As these two subtypes can be histologically similar, the morphometry plays a minor role, while metabolite data are of higher importance for classification. Hence, fewer morphometric features are ranked among the top 50 (40%), but they are still beneficial for tumour subtype classification. The synergistic effect is reflected by the high ranking of morphometric features within the classifier (Figure 3F).

Even though morphometric data are readily available in any MSI experiment, it has not been exploited to improve the predictive quality of molecular classifiers so far. This study provides evidence that the synergy of morphometric and molecular data improves renal carcinoma subtyping. Our study was performed on a large patient cohort (*n* = 853) and on clinically relevant FFPE tissue samples using metabolite data. The classifier trained on the combined data set or morphometric and metabolite data outperformed the classifiers trained on the individual data sets for each tumour subtype and reached an accuracy of 88.04%. Finally, the classifier was trained on chRCC and RO – two tumour subtypes that are sometimes difficult to distinguish based on histology alone – and was able to distinguish the subtypes with high accuracy (91%). In conclusion, we propose to utilize the so far unrecognized potential and synergy of computer‐aided image analysis and spatial metabolomics – both types of data available in all MSI experiments – to improve AI‐based diagnostics and tumour subtyping in general.

## CONFLICT OF INTEREST

The authors declare no conflict of interest.

## Supporting information

Supporting Information

## References

[1] Bergmann L , Weber S , Hartmann A , Ahrens M . Pathology and systemic therapy of non‐clear cell renal cell carcinoma: an overview. Expert Rev Anticancer Ther. 2021;21:1273‐1286. 34291700 10.1080/14737140.2021.1959319

[2] Kriegsmann M , Casadonte R , Maurer N , Stoehr C , Erlmeier F , Moch H . Mass spectrometry imaging differentiates chromophobe renal cell carcinoma and renal oncocytoma with high accuracy. J Cancer. 2020;11:6081‐6089. 32922548 10.7150/jca.47698 PMC7477404

[3] Ohashi R , Martignoni G , Hartmann A , et al. Multi‐institutional re‐evaluation of prognostic factors in chromophobe renal cell carcinoma: proposal of a novel two‐tiered grading scheme. Virchows Arch. 2020;476:409‐418. 31760491 10.1007/s00428-019-02710-w

[4] Erlmeier F , Feuchtinger A , Borgmann D , et al. Supremacy of modern morphometry in typing renal oncocytoma and malignant look‐alikes. Histochem Cell Biol. 2015;144:147‐156. 25929744 10.1007/s00418-015-1324-4

[5] Mazal PR , Exner M , Haitel A , et al. Expression of kidney‐specific cadherin distinguishes chromophobe renal cell carcinoma from renal oncocytoma. Hum Pathol. 2005;36:22‐28. 15712178 10.1016/j.humpath.2004.09.011

[6] Schöne C , Höfler H , Walch A . MALDI imaging mass spectrometry in cancer research: combining proteomic profiling and histological evaluation. Clin Biochem. 2013;46:539‐545. 23388677 10.1016/j.clinbiochem.2013.01.018

[7] Prade VM , Kunzke T , Feuchtinger A , et al. De novo discovery of metabolic heterogeneity with immunophenotype‐guided imaging mass spectrometry. Mol Metab. 2020;36:100953. 32278304 10.1016/j.molmet.2020.01.017 PMC7149754

[8] Ščupáková K , Dewez F , Walch AK , Heeren RMA , Balluff B . Morphometric cell classification for single‐cell MALDI‐mass spectrometry imaging. Angew Chemie. 2020;132:17600‐17603. 10.1002/anie.202007315 PMC7540554 32668069

[9] Neumann EK , Djambazova KV , Caprioli RM , Spraggins JM . Multimodal imaging mass spectrometry: next generation molecular mapping in biology and medicine. J Am Soc Mass Spectrom. 2020;31:2401‐2415. 32886506 10.1021/jasms.0c00232 PMC9278956

[10] Huber K , Kunzke T , Buck A , et al. Multimodal analysis of formalin‐fixed and paraffin‐embedded tissue by MALDI imaging and fluorescence in situ hybridization for combined genetic and metabolic analysis. Lab Investig. 2019;99:1535‐1546. 31148595 10.1038/s41374-019-0268-z

[11] Vijayalakshmi K , Shankar V , Bain RM , et al. Identification of diagnostic metabolic signatures in clear cell renal cell carcinoma using mass spectrometry imaging. Int J Cancer. 2020;147:256‐265. 31863456 10.1002/ijc.32843 PMC8571954

[12] Tamura K , Horikawa M , Sato S , Miyake H , Setou M . Discovery of lipid biomarkers correlated with disease progression in clear cell renal cell carcinoma using desorption electrospray ionization imaging mass spectrometry. Oncotarget. 2019;10:1688‐1703. 30899441 10.18632/oncotarget.26706 PMC6422196

[13] Zhang J , Li SQ , Lin JQ , Yu W , Eberlin LS . Mass spectrometry imaging enables discrimination of renal oncocytoma from renal cell cancer subtypes and normal kidney tissues. Cancer Res. 2020;80:689‐698. 31843980 10.1158/0008-5472.CAN-19-2522 PMC7024663

[14] Kriegsmann M , Casadonte R , Maurer N , et al. Mass spectrometry imaging differentiates chromophobe renal cell carcinoma and renal oncocytoma with high accuracy. J Cancer. 2020;11:6081‐6089. 32922548 10.7150/jca.47698 PMC7477404

[15] Lu HC , Patterson NH , Judd AM , Reyzer ML , Sehn JK . Imaging mass spectrometry is an accurate tool in differentiating clear cell renal cell carcinoma and chromophobe renal cell carcinoma: a proof‐of‐concept study. J Histochem Cytochem. 2020;68:403‐411. 32466698 10.1369/0022155420931417

