# Supplementary material for: The synergism of spatial metabolomics and morphometry improves machine learning‐based renal tumour subtype classification
Source: Clin Transl Med. 2022 Feb 20;12(2):e666. doi: 10.1002/ctm2.666 (PMC8858620; doi:10.1002/ctm2.666)

Supplementary Information

The synergism of spatial metabolomics and morphometry improves machine learning based renal tumour subtype classification

Verena M. Prade, Na Sun, Jian Shen, Annette Feuchtinger, Thomas Kunzke, Achim Buck, Peter Schraml, Holger Moch, Kristina Schwamborn, Michael Autenrieth, Jürgen E. Gschwend, Franziska Erlmeier, Arndt Hartmann, and Axel Walch

Patient tissues

Formalin fixed paraffin embedded (FFPE) renal tumour samples comprising 552 clear cell renal cell carcinoma (ccRCC), 122 papillary renal cell carcinoma (pRCC), 108 chromophobe renal cell carcinoma (chRCC) and 71 renal oncocytoma (RO) were collected from the archives of the Department of Pathology and Molecular Pathology of the University Hospital Zurich (1993-2013) and of the Technical University of Munich (1996-2014). Tissue microarrays (TMAs) were constructed as described [1,2]. Tissue cylinders with 0.6 mm (Zurich) and 1.0 mm (Munich) diameter were punched from morphologically representative regions of paraffin donor blocks.

The study was approved by the Cantonal Ethics Committee of Zurich (BASEC-No_2019-01959) and the Ethics Committee of the Technical University of Munich (384/13) in accordance with the Swiss/German Human Research Act and with the Declaration of Helsinki.

**MALDI mass spectrometry imaging**

Tissue preparation steps for the high mass resolution matrix-assisted laser desorption/ionization (MALDI) fourier-transform ion cyclotron resonance (FT-ICR) mass spectrometry imaging analysis was performed as previously described [3,4]. In brief, FFPE TMAs were sectioned with 4 µm (Microm, HM340E, Thermo Fisher Scientific, USA) and mounted onto indium-tin-oxide (ITO)-coated glass slides (Bruker Daltonik, Bremen, Germany) pretreated with 1:1 poly-L-lysine (Sigma Aldrich, Munich, Germany) and 0.1% Nonidet P-40 (Sigma-Aldrich). Prior to MALDI matrix application, 10 mg/ml 9-aminoacridine hydrochloride monohydrate matrix (Sigma-Aldrich) in 70% methanol, FFPE sections were adhered by incubating the slide for 1 h at 70°C, deparaffinized in xylene (2 x 8 min), and air-dried. Spray-coating of the matrix was conducted using the SunCollectTM sprayer (Sunchrom, Friedrichsdorf, Germany) in eight passes (ascending flow rates 10 µl/min, 20 µl/min, 30 µl/min for layers 1-3, and layers 4-8 with 40 µl/min), utilizing 2 mm line distance and a spray velocity of 900 mm/min.

Metabolites were detected in negative-ion mode on a 7 T Solarix XR FT-ICR mass spectrometer (Bruker Daltonik) equipped with a dual ESI-MALDI source and a SmartBeam-II Nd: YAG (355 nm) laser. Data acquisition parameters were specified in ftmsControl software 2.2 and flexImaging (v. 5.0) (Bruker Daltonik). Mass spectra were acquired in negative-ion mode covering m/z 75-1,000. For internal mass calibration, the 9-AA matrix ion signal (m/z 193.0771) was used as lock mass minimizing scan-to-scan (pixel-to-pixel) variations during the MALDI measurement. The laser operated at a frequency of 1,000 Hz utilizing 200 laser shots per pixel with a pixel resolution of 60 µm. External calibration of the instrument was performed with L-Arginine in the ESI mode.

MALDI mass spectra were root mean square normalized with SCiLS (v. 2020b Pro) and picked peaks were exported as imzML files for further data processing and subsequent analysis with the SPACiAL pipeline [5]. Hematoxylin & eosin (H&E) stainings of the same tissue sections were registered to the MALDI data and regions of interest (tumour) were annotated based on these stainings. The MALDI mass spectra were then root mean square normalized with SCiLS (v. 2020b Pro) and exported. Peak picking and image co-registration was performed pixel-wise as previously described [5]. Briefly, for peak picking, the Bruker software SCiLS (v. 2020b Pro) was used to export all root mean square normalized mass spectra as processed imzML files. Pixel-wise and parallelized peak picking were performed and resampled with an in-house python 3 pipeline. Noise levels were estimated for windows of 10 Da, and all peaks falling below their respective noise level were filtered. Finally, peaks within each spectrum were merged and aligned. For image co-registration, the imzML file of picked peaks was used to create a master image of the MALDI measurement region (imzML-grid), allowing an exact integration and correlation of molecular MALDI data with morphology data. The integration of mass spectra and image data is done by co-registering the subsequently scanned tissue to MALDI imaging mass spectrometry and mapping the matrix ablation marks to the imzML-grid. For classifier training and evaluation, regions of interest (tumour) were annotated based on H&E stainings. Mean peak intensities of tumour regions were calculated for each patient and used for classifier training. Peak annotation was performed using HMDB [6] and KEGG [7] databases, while allowing M-H, M-H2O, M+K-2H, M+Na-2H, and M+Cl as negative adducts with a mass tolerance of 4 ppm.

**Morphometrics analysis**

Consecutive H&E stained tissue sections were scanned with an AxioScan.Z1 digital slide scanner (Zeiss, Jena, Germany) equipped with a 20x magnification objective. Images were morphometrically evaluated using the image analysis software Definiens Developer XD 2 (Definiens AG, Munich, Germany) following a previously published procedure [2]. A description of the morphometric attributes for tissue or cell compartment colour, shape, size etc. is shown in Supplementary Table 1.

**Classifier training and evaluation**

Morphometric (n = 110) and metabolomic features (n = 2111) were used for classifier training. While training the classifiers, it was ensured that the validation sets remained completely independent from the training data. In 200 repetitions, the patients were randomly split into a training (2/3) and an independent validation set (1/3). In each repetition, the data was normalized for training and validation set, separately. Feature selection was done by calculating a Kruskal-Wallis test with subsequent Benjamini-Hochberg correction (Python 3.7, ‘scipy’ v.1.5.2 and ‘statsmodels’ v.0.11.1, *P*< 0.01) on the training sets. Similarly, parameter optimization techniques on each training set separately were tested but not applied in the final training, since they did not improve the performance of the classifier, but instead led to overfitting. The random forest classifier itself was trained using the Python 3.7 ‘sklearn’ (v. 0.22.1) package.

The performance of the classifiers was compared by calculating the mean accuracy, as well as the precision, recall and F1-score. Precision is calculated by dividing the number of true positives by the sum of the true positives and false positives. Recall (i.e. sensitivity) is calculated by dividing the number of true positives by the sum of the true positives and false negatives. The F1-score is the harmonic mean of the precision and recall. All values were multiplied with 100 to represent percentages. Finally, the importance of a feature is computed as the (normalized) total reduction of the criterion brought by that feature (Gini importance).

1. Dahinden, C.; Ingold, B.; Wild, P.; Boysen, G.; Luu, V.D.; Montani, M.; Kristiansen, G.; Sulser, T.; Bühlmann, P.; Moch, H.; et al. Mining tissue microarray data to uncover combinations of biomarker expression patterns that improve intermediate staging and grading of clear cell renal cell cancer. *Clin. Cancer Res.* **2010**, *16*, 88–98, doi:10.1158/1078-0432.CCR-09-0260.

2. Erlmeier, F.; Feuchtinger, A.; Borgmann, D.; Rudelius, M.; Autenrieth, M.; Walch, A.K.; Weirich, G. Supremacy of modern morphometry in typing renal oncocytoma and malignant look-alikes. *Histochem. Cell Biol.* **2015**, *144*, 147–156, doi:10.1007/s00418-015-1324-4.

3. Buck, A.; Ly, A.; Balluff, B.; Sun, N.; Gorzolka, K.; Feuchtinger, A.; Janssen, K.P.; Kuppen, P.J.K.; Van De Velde, C.J.H.; Weirich, G.; et al. High-resolution MALDI-FT-ICR MS imaging for the analysis of metabolites from formalin-fixed, paraffin-embedded clinical tissue samples. *J. Pathol.* **2015**, *237*, 123–132, doi:10.1002/path.4560.

4. Ly, A.; Buck, A.; Balluff, B.; Sun, N.; Gorzolka, K.; Feuchtinger, A.; Janssen, K.P.; Kuppen, P.J.K.; Van De Velde, C.J.H.; Weirich, G.; et al. High-mass-resolution MALDI mass spectrometry imaging of metabolites from formalin-fixed paraffin-embedded tissue. *Nat. Protoc.* **2016**, *11*, 1428–1443, doi:10.1038/nprot.2016.081.

5. Prade, V.M.; Kunzke, T.; Feuchtinger, A.; Rohm, M.; Luber, B.; Lordick, F.; Buck, A.; Walch, A. De novo discovery of metabolic heterogeneity with immunophenotype-guided imaging mass spectrometry. *Mol. Metab.* **2020**, *36*, 100953, doi:10.1016/j.molmet.2020.01.017.

6. Wishart, D.S.; Feunang, Y.D.; Marcu, A.; Guo, A.C.; Liang, K.; Rosa, V.; Sajed, T.; Johnson, D.; Li, C.; Karu, N.; et al. HMDB 4.0 : the human metabolome database for 2018. *Nucleic Acids Res.* **2018**, *46*, 608–617, doi:10.1093/nar/gkx1089.

7. Kanehisa, M. KEGG: Kyoto Encyclopedia of Genes and Genomes. *Nucleic Acids Res.* **2000**, *28*, 27–30, doi:10.1093/nar/28.1.27.

Supplementary Table 1: Morphometric features describing density, area, colour, shape or compactness of regions of interest. Segments 1 and 2 are regions of interest created by the Definiens software based on colour channels.

| **identifier** | **feature type** | **description** |
| --- | --- | --- |
| morph_1 | density | cell density |
| morph_2 | area | avg. cell area |
| morph_3 | colour | avg. brownness (cell) |
| morph_4 | colour | avg. brownness (cytoplasm) |
| morph_5 | colour | avg. nucleus brownness / avg. cytoplasm brownness |
| morph_6 | area | avg. area (nucleus) |
| morph_7 | area | std. dev. area (nucleus) |
| morph_8 | shape | avg. roundness (nucleus) |
| morph_9 | shape | std. dev. roundness (nucleus) |
| morph_10 | compactness | avg. compactness (nucleus) |
| morph_11 | compactness | std. dev. compactness (nucleus) |
| morph_12 | shape | avg. shape index (nucleus) |
| morph_13 | shape | std. dev. shape index (nucleus) |
| morph_14 | colour | avg. layer 1 - red (nucleus) |
| morph_15 | colour | std. dev. layer 1 - red (nucleus) |
| morph_16 | colour | avg. layer -green (nucleus) |
| morph_17 | colour | std. dev. layer 1 - green (nucleus) |
| morph_18 | colour | avg. layer 1 - blue (nucleus) |
| morph_19 | colour | std. dev. layer 1 - blue (nucleus) |
| morph_20 | shape | avg. length/width (nucleus) |
| morph_21 | shape | std. dev. length/width (nucleus) |
| morph_22 | density | avg. density (nucleus) |
| morph_23 | density | std. dev. density (nucleus) |
| morph_24 | shape | avg. elliptic fit (nucleus) |
| morph_25 | shape | std. dev. elliptic Fit (nucleus) |
| morph_26 | colour | avg. brownness (nucleus) |
| morph_27 | colour | std. dev. brownness (nucleus) |
| morph_28 | colour | avg. hematoxylin intensity (nucleus) |
| morph_29 | colour | std. dev. hematoxylin intensity (nucleus) |
| morph_30 | density | avg. optical density (nucleus) |
| morph_31 | density | std. dev. optical density (nucleus) |
| morph_32 | shape | avg. circularity (nucleus) |
| morph_33 | shape | std. dev. circularity (nucleus) |
| morph_34 | shape | avg. ellipticity (nucleus) |
| morph_35 | shape | std. dev. ellipticity (nucleus) |
| morph_36 | colour | avg. layer 1 - red (cytoplasm) |
| morph_37 | colour | std. dev. layer 1 - red (cytoplasm) |
| morph_38 | colour | avg. layer - green (cytoplasm) |
| morph_39 | colour | std. dev. layer 1 - green (cytoplasm) |
| morph_40 | colour | avg. layer 1 - blue (cytoplasm) |
| morph_41 | colour | std. dev. layer 1 - blue (cytoplasm) |
| morph_42 | colour | avg. brownness (cytoplasm) |
| morph_43 | colour | std. dev. brownness (cytoplasm) |
| morph_44 | colour | avg. hematoxylin intensity (cytoplasm) |
| morph_45 | colour | std. dev. hematoxylin intensity (cytoplasm) |
| morph_46 | density | avg. optical density (cytoplasm) |
| morph_47 | density | std. dev. optical density (cytoplasm) |
| morph_48 | area | avg. area (segment 1) |
| morph_49 | area | std. dev. area (segment 1) |
| morph_50 | shape | avg. roundness (segment 1) |
| morph_51 | shape | std. dev. roundness (segment 1) |
| morph_52 | compactness | avg. compactness (segment 1) |
| morph_53 | compactness | std. dev. compactness (segment 1) |
| morph_54 | shape | avg. shape index (segment 1) |
| morph_55 | shape | std. dev. shape index (segment 1) |
| morph_56 | colour | avg. layer 1 - red (segment 1) |
| morph_57 | colour | std. dev. layer 1 - red (segment 1) |
| morph_58 | colour | avg. layer - green (segment 1) |
| morph_59 | colour | std. dev. layer 1 - green (segment 1) |
| morph_60 | colour | avg. layer 1 - blue (segment 1) |
| morph_61 | colour | std. dev. layer 1 - blue (segment 1) |
| morph_62 | shape | avg. length/width (segment 1) |
| morph_63 | shape | std. dev. length/width (segment 1) |
| morph_64 | density | avg. density (segment 1) |
| morph_65 | density | std. dev. density (segment 1) |
| morph_66 | shape | avg. elliptic fit (segment 1) |
| morph_67 | shape | std. dev. Elliptic fit (segment 1) |
| morph_68 | colour | avg. brownness (segment 1) |
| morph_69 | colour | std. dev. brownness (segment 1) |
| morph_70 | colour | avg. hematoxylin intensity (segment 1) |
| morph_71 | colour | std. dev. hematoxylin intensity (segment 1) |
| morph_72 | density | avg. optical density (segment 1) |
| morph_73 | density | std. dev. optical density (segment 1) |
| morph_74 | shape | avg. circularity (segment 1) |
| morph_75 | shape | std. dev. circularity (segment 1) |
| morph_76 | shape | avg. ellipticity (segment 1) |
| morph_77 | shape | std. dev. ellipticity (segment 1) |
| morph_78 | area | avg. area (segment 2) |
| morph_79 | area | std. dev. area (segment 2) |
| morph_80 | shape | avg. roundness (segment 2) |
| morph_81 | shape | std. dev. roundness (segment 2) |
| morph_82 | compactness | avg. compactness (segment 2) |
| morph_83 | compactness | std. dev. compactness (segment 2) |
| morph_84 | shape | avg. shape index (segment 2) |
| morph_85 | shape | std. dev. shape index (segment 2) |
| morph_86 | colour | avg. layer 1 - red (segment 2) |
| morph_87 | colour | std. dev. layer 1 - red (segment 2) |
| morph_88 | colour | avg. layer - green (segment 2) |
| morph_89 | colour | std. dev. layer 1 - green (segment 2) |
| morph_90 | colour | avg. layer 1 - blue (segment 2) |
| morph_91 | colour | std. dev. layer 1 - blue (segment 2) |
| morph_92 | shape | avg. length/width (segment 2) |
| morph_93 | shape | std. dev. length/width (segment 2) |
| morph_94 | density | avg. density (segment 2) |
| morph_95 | density | std. dev. density (segment 2) |
| morph_96 | shape | avg. elliptic fit (segment 2) |
| morph_97 | shape | std. dev. elliptic fit (segment 2) |
| morph_98 | colour | avg. brownness (segment 2) |
| morph_99 | colour | std. dev. brownness (segment 2) |
| morph_100 | colour | avg. hematoxylin intensity (segment 2) |
| morph_101 | colour | std. dev. hematoxylin intensity (segment 2) |
| morph_102 | density | avg. optical density (segment 2) |
| morph_103 | density | std. dev. optical density (segment 2) |
| morph_104 | shape | avg. circularity (segment 2) |
| morph_105 | shape | std. dev. circularity (segment 2) |
| morph_106 | shape | avg. ellipticity (segment 2) |
| morph_107 | shape | std. dev. ellipticity (segment 2) |
| morph_116 | colour | avg. brownness (ROI) |
| morph_117 | colour | avg. hematoxylin intensity (ROI) |
| morph_118 | density | avg. optical density (ROI) |

Supplementary Figure 1: Example ion images of the top features in the classifier. A. glycerol 3-phosphate (m/z 152.9957), B. Hexose phosphate (m/z 259.0224), C. Oleic acid (m/z 281.2487). Each column indicates mean with standard error. P values are corresponding to the Kruskal–Wallis test.


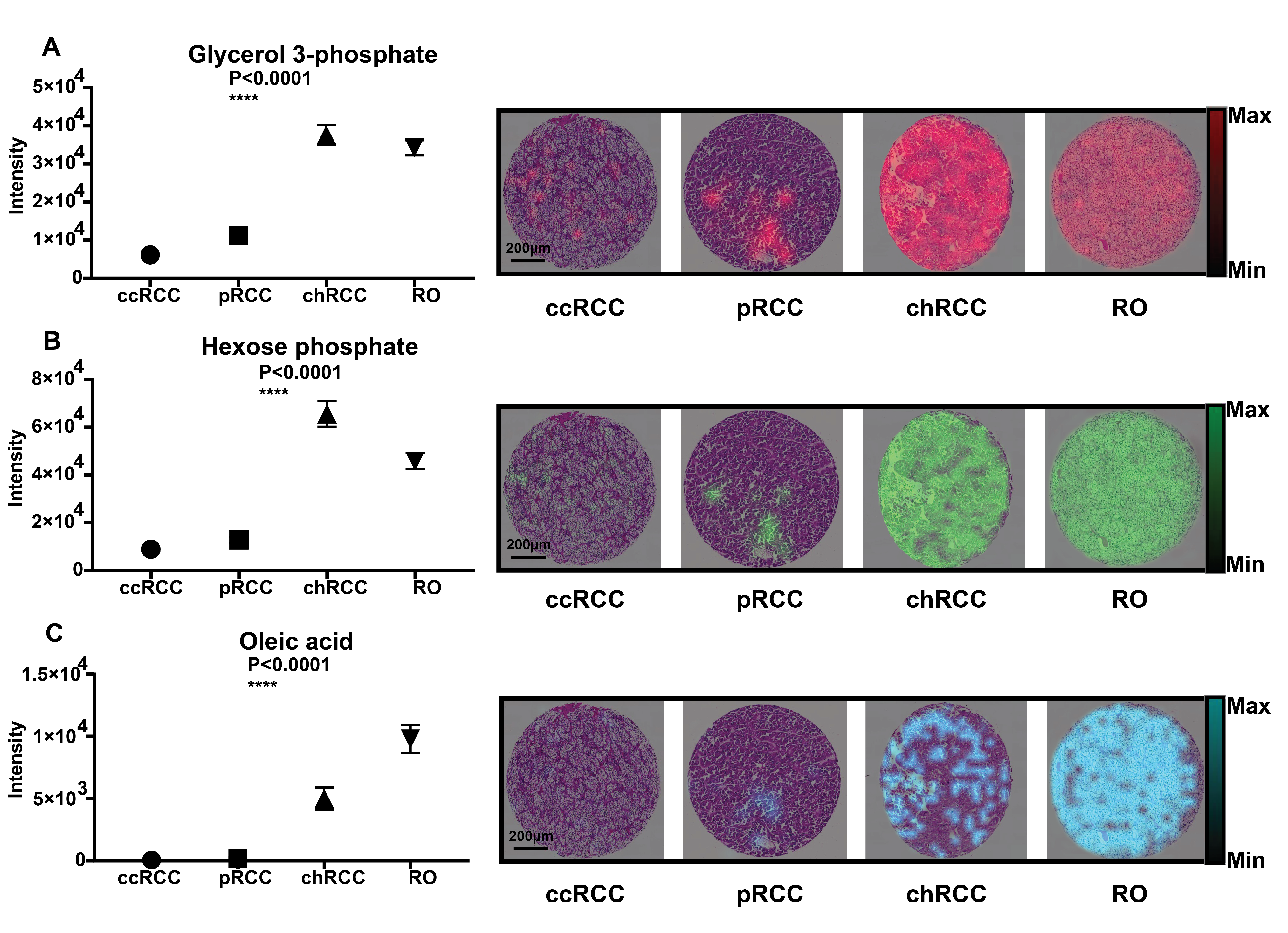

Supplement: Supplementary file 1 — Supporting Information [file CTM2-12-e666-s001.docx]
